# Structural basis of homologous recombination

**DOI:** 10.1007/s00018-019-03365-1

**Published:** 2019-11-20

**Authors:** Yueru Sun, Thomas J. McCorvie, Luke A. Yates, Xiaodong Zhang

**Affiliations:** Section of Structural Biology, Department of Infectious Diseases, Imperial College, London, SW7 2AZ UK

**Keywords:** Homologous recombination, Cryo electron microscopy, X-ray crystallography, Double-strand break repair, DNA damage signalling and repair

## Abstract

Homologous recombination (HR) is a pathway to faithfully repair DNA double-strand breaks (DSBs). At the core of this pathway is a DNA recombinase, which, as a nucleoprotein filament on ssDNA, pairs with homologous DNA as a template to repair the damaged site. In eukaryotes Rad51 is the recombinase capable of carrying out essential steps including strand invasion, homology search on the sister chromatid and strand exchange. Importantly, a tightly regulated process involving many protein factors has evolved to ensure proper localisation of this DNA repair machinery and its correct timing within the cell cycle. Dysregulation of any of the proteins involved can result in unchecked DNA damage, leading to uncontrolled cell division and cancer. Indeed, many are tumour suppressors and are key targets in the development of new cancer therapies. Over the past 40 years, our structural and mechanistic understanding of homologous recombination has steadily increased with notable recent advancements due to the advances in single particle cryo electron microscopy. These have resulted in higher resolution structural models of the signalling proteins ATM (ataxia telangiectasia mutated), and ATR (ataxia telangiectasia and Rad3-related protein), along with various structures of Rad51. However, structural information of the other major players involved, such as BRCA1 (breast cancer type 1 susceptibility protein) and BRCA2 (breast cancer type 2 susceptibility protein), has been limited to crystal structures of isolated domains and low-resolution electron microscopy reconstructions of the full-length proteins. Here we summarise the current structural understanding of homologous recombination, focusing on key proteins in recruitment and signalling events as well as the mediators for the Rad51 recombinase.

## Introduction

In eukaryotic cells, there are two major processes that act to repair double-strand breaks (DSB): end-joining (EJ) and homologous recombination (HR). Processes that involve end-joining are template independent and can be subdivided further into non-homologous EJ and microhomology-mediated EJ. End-joining (or more accurately ligation) without a template, or sister chromatid, can result in the loss of genetic material or chromosomal rearrangements when a large number of DSBs occur [1]. Homologous recombination, on the other hand, requires a DNA template for the repair process and is therefore considered more accurate as any missing genetic information lost in the DSB or during end processing is recovered. Similarly to end-joining, recombination can also be subdivided into distinct pathways known broadly as Single-Stranded Annealing (SSA), synthesis-dependent strand annealing (SDSA), break-induced replication (BIR) and canonical DSB Repair. In this review we focus on current structural insights into key proteins involved in canonical DSB repair via homologous recombination.

## Overview of DNA damage response signal transduction

**Fig. 1:**
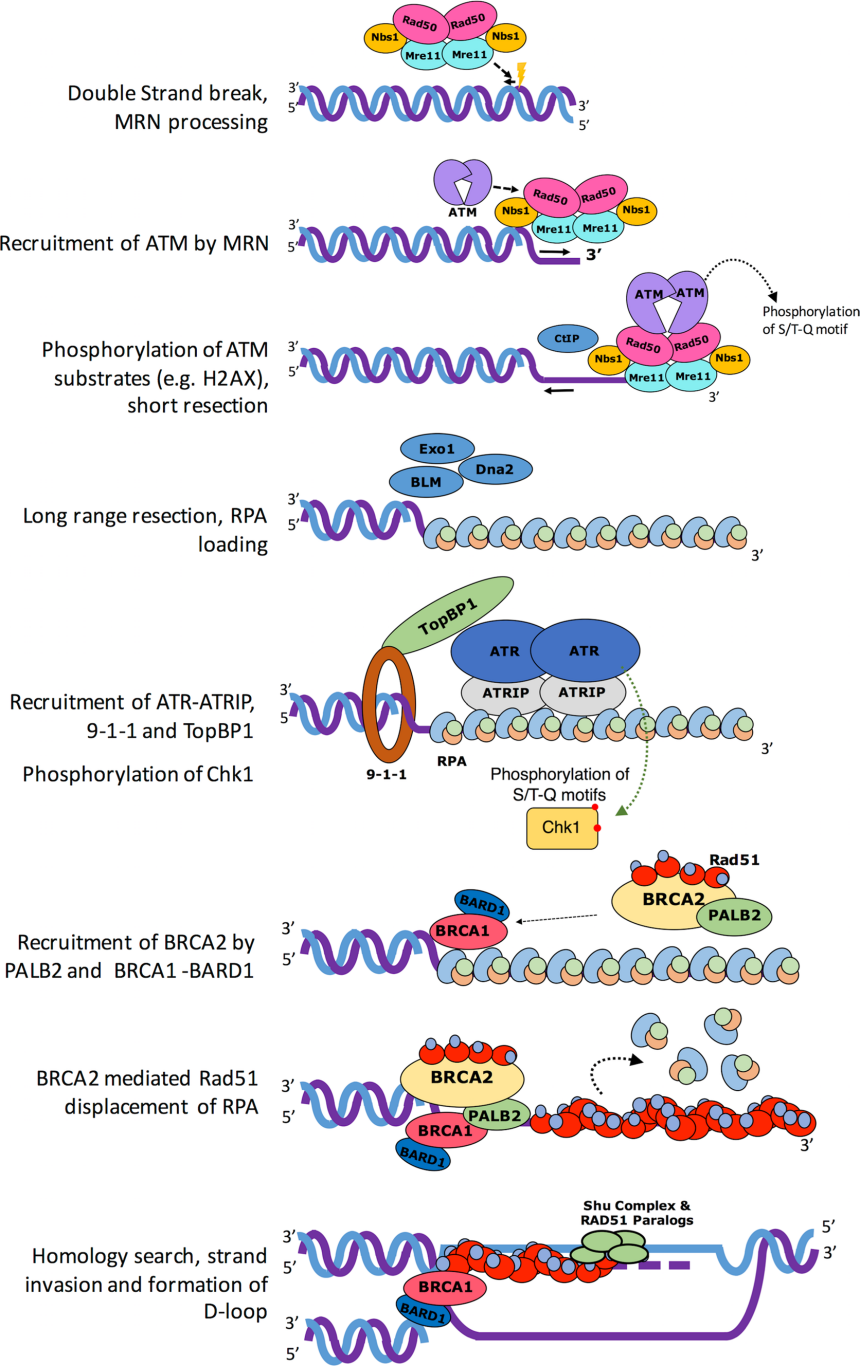
Brief overview of DDR signalling. Canonical double-strand break (DSB) DNA damage response signalling pathway (see text for details)

Eukaryotes have evolved sophisticated and highly organised processes in response to DNA damage. This coordinated effort is known as the DNA damage response (DDR) and operates to sense and signal genotoxic events, which are subsequently resolved by DNA repair machineries or cell death if DNA remains unrepaired. The DDR signal transduction pathway is primarily mediated by Serine/Threonine protein kinases belonging to the phosphatidylinositol 3-kinase-like protein kinase (PIKK) family, which include ATM (Ataxia Telangiectasia Mutated), ATR (ATM-related) and DNA-PK (DNA-dependent protein kinase). ATM and DNA-PK are recruited to a DNA double-strand break [2], with the activation of each kinase directing distinct repair pathways. For instance, DNA-PK regulates proteins involved in DSB end joining (NHEJ), whereas ATM regulates hundreds of substrates that ultimately bring about HR and cell cycle arrest. Whilst ATM responds to DSB, ATR, along with its partner ATRIP (ATR-interacting protein), is activated after its recruitment to replication protein A (RPA)-coated single-stranded DNA (ssDNA), generated at stalled replication forks or as intermediates during the processing of DSB [3] (Fig. 1). ATM and ATR phosphorylate serine/threonine residues of hundreds of substrate proteins at S/T-Q motifs and can elicit a second wave of phosphorylation events via their activation of other kinases such as Chk1 and Chk2, for example. DNA-PK catalytic subunit (DNA-PKcs) also shows preferences for phosphorylating serine/threonines residues at S/T-Q motifs. Additional residues in the vicinity of the S/T-Q motifs have been shown to be determinants of phosphorylation, such as hydrophobic or acidic amino acids promoting phosphorylation, whereas basic residues are negative determinants. Thus, the ATM, ATR and DNA-PK kinases have overlapping targets and can be considered ‘master transducers’ of the DDR [4].

In the HR pathway, the signalling of a DSB is initiated via the binding of the MRN complex (Mre11, Rad50 and Nbs1) to the broken DNA ends. The MRN complex plays a critical role in recruiting and activating ATM at DSB sites (Fig. 1). Once activated, ATM is able to phosphorylate a variety of proteins involved in the execution of the DDR, for example histone H2AX [5], where its phosphorylated form serves as a platform for further recruitment of additional DDR factors around the break site. DSBs also activate ATM yeast orthology Tel1 kinase signalling [6], with both ATM and ATR being critical for DSB repair and checkpoint activation. ATR signalling is elicited whenever ssDNA is generated, and this is reflected in ATR’s roles in a broad range of DNA damage, particularly those that interfere with DNA replication. Thus ATM and ATR kinases have DNA damage specificities and non-redundant functions despite overlapping substrates [7].

## Architecture of the MRN complex: the initiator of the DSB repair

The MRN complex initiates DSB repair by recognising and resecting the free DNA ends at damaged sites. The complex has a multitude of functions: (1) facilitating ATP-dependent endonucleolytic cleavage near the double stand break and short range 3′–5′ resection from the nick site towards the DNA end to generate a 3′ overhang, (2) promoting recruitment of endonuclease ExoI or BLM complex to perform bulk 5′–3′ DNA resection, (3) loading of RPA on ssDNA overhang and activation of ATM (Fig. 1) [8–10]. A recent study suggests that MRN also acts as a processive factor for ExoI during long range resection [10]. In addition to its prominent roles in HR as the primary recruitment and activation factor for ATM, MRN can also stimulate NHEJ in an ATM-independent manner [11]. Furthermore, studies suggest recruitment of Cdk2 phosphorylated CtIP and BRCA1 at G2/S phase to MRN facilitates the removal of Ku70-Ku80 (Ku) cap at DNA end and promotes the dephosphorylation of 53BP1 [12], which in turn simulates HR and inhibit NHEJ [13, 14].

**Fig. 2:**
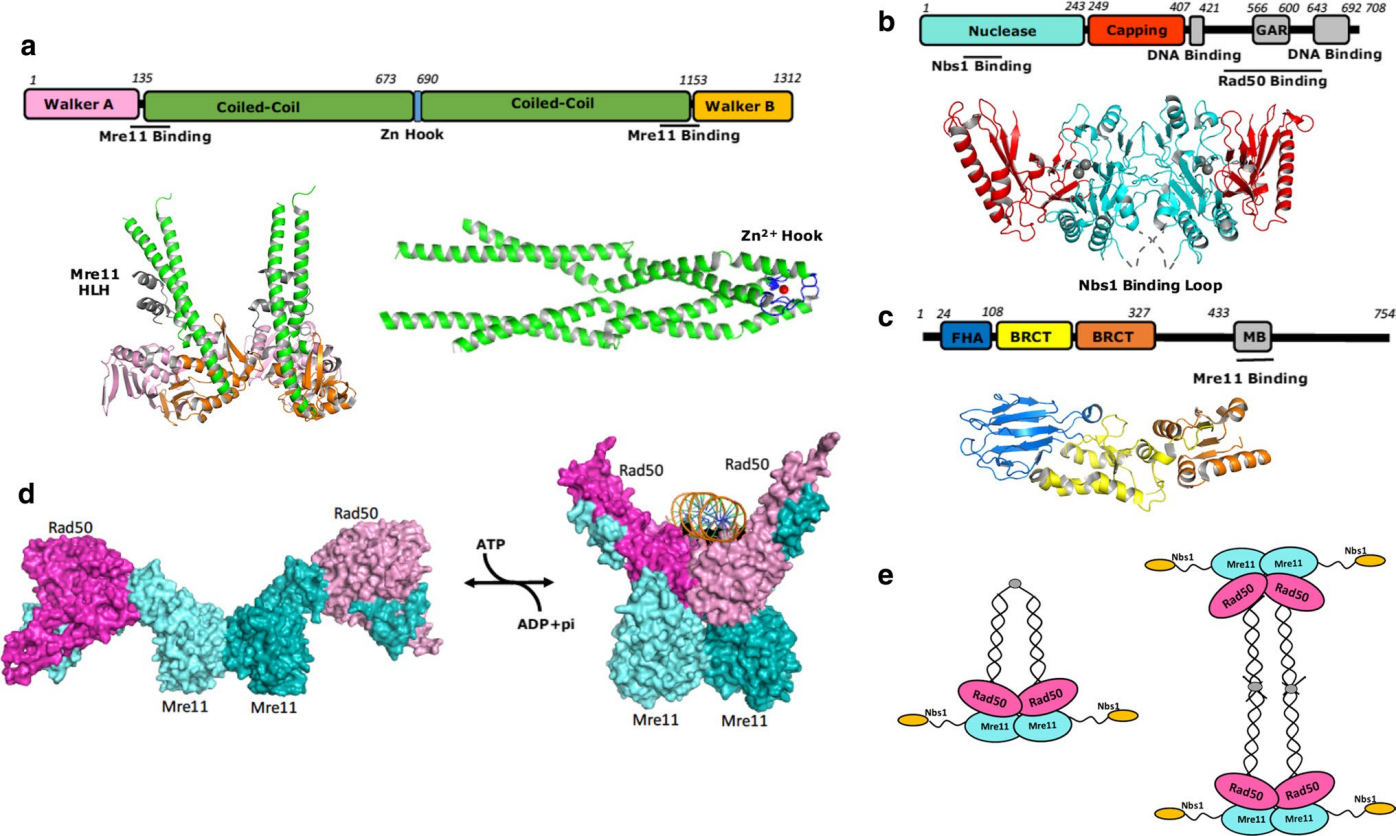
Structures of the MRN complex. **a** Domain structure of human Rad50, along with crystal structure of *Pyrococcus furiosus* RAD50 ATPase dimer (left, PDB ID: 3QKR) and human RAD50 coiled-coil domain (right, PDB ID: 5GOX) The N-terminal Walker A domain is shown in pink, C-terminal Walker B domain in orange, coiled-coil domain in green and Zn-hook in blue. Zn^2+^ is indicated as a red sphere. Bound Mre11 helix-loop-helix is shown in violet. **b** Domain structure of human Mre11 and crystal structure of human Mre11 N-terminal domain (PDB ID: 3T1I). Nuclease domain is shown in cyan, capping domain in red. Mn^2+^ is indicated as grey spheres. The latching loops that bind Nbs1 are disordered in this structure and are annotated as grey dash line. **c** Domain structure of human Nbs1 and crystal structure of *Schizosaccharomyces pombe* Nbs1 (PDB ID: 3HUF) with FHA domain shown in blue, BRCT 1 shown in yellow and BRCT 2 in orange. **d** Surface representation of ATP-hydrolysis driven RAD50 dimerisation (PDB ID: 5F3 W for the close conformation and 4FBW for the open conformation). **e** Intramolecular and intermolecular complex forms of MRN

Mre11 and Rad50 are evolutionarily conserved enzymes. Mre11-Rad50 complexes found in archaea and bacteria share the same enzymatic activities and morphologies as their human counterpart, whereas Nbs1 only shows homology in eukaryotes [15]. Early atomic force microscopy (AFM) and electron microscopy studies on human and *Saccharomyces cerevisiae* Mre11-Rad50 reveal a heterodimeric architecture comprising of two Mre11 and two Rad50 molecules. As a structural maintenance of chromosomes (SMC) family protein, Rad50 has a nucleotide-binding head domain and a long anti-parallel coiled-coil insertion (Fig. 2a). Two Rad50 head domains are brought together by two long, 15–50 nm coiled-coils with Zn^2+^ hook domain dimerising at the middle hinge [16, 17] (Fig. 2a). Mre11 also dimerises and attaches to the globular domains of Rad50 through a helix-loop-helix (HLH) motif to form a globular head that binds DNA end [18–20]. Mre11 is composed of two α/β domains, an N-terminal Mn^2+^ bound nuclease domain and a C-terminal capping domain responsible for substrate recognition (Fig. 2b) [21, 22].

Nbs1 attaches to Mre11 through two binding motifs, a Mre11 binding (MB) motif and a highly conserved FKXXFXK motif, which binds across the Mre11 dimer to the eukaryotic specific insertion loops on both Mre11 molecules [23]. Although the stoichiometry of Nbs1 to Mre11 is 2:2, only one FKXXFXK motif of the two Nbs1 is involved in Mre11 interface, sterically occluding the other Nbs1 [23]. The N-terminal domain of Nbs1 extends away from the globular head of Mre11-Rad50 and consists of a canonical fork head associated domain (FHA) and tandem BRCT repeats that are able to accommodate diverse phosphorylated proteins (Fig. 2c). The crystal structure of *S. pombe* Nbs1 shows that a 20° rotation of BRCT2 towards BRCT1-BRCT2 interface is triggered by bound CHK-dependent phosphorylated Ctp1, an orthologue of human CtIP [24]. As a result, the N-terminal domain of Nbs1 shifts 10 Å closer towards the C-terminus and possibly, the Mre11-Rad50 core. These structural transitions are likely to influence Mre11 and Rad50 in a regulatory manner [23–26]. However, structural evidence of the interplay between Nbs1 and the core of the MR complex, to facilitate regulation, remains unclear.

Crystal structures of ATP analogue-bound Mre11-Rad50 in complex with double-strand DNA (dsDNA) and recent cryoEM structure of the E. coli Mre11-Rad50 homolog in complex with DNA reveal an ATP-dependent clamp-like mechanism (Fig. 2d) [27–30]. Upon ATP binding, the two Rad50 head domains close up to facilitate the binding of DNA. Upon ATP hydrolyses, the Rad50 dimeric head domains dissociate, exposing the nuclease site of Mre11 to carry out DNA resection [28–32]. However, how the ATP-dependent open-close transition is regulated upon DSB recognition, repair initiation and downstream signalling transduction is still not well understood. A recent solution NMR study characterising fast timescale dynamics on side chain methyl groups of valines and isoleucines in Rad50 hinge helices adjacent to the Mre11-Rad50 interface reveals that ATP hydrolysis and Rad50 dimerisation are coupled through a dynamic allosteric network [27, 33]. Although only cause subtle structural changes, mutations disrupting the allosteric pathway increase the activity of ATP hydrolysis and the propensity for dimerization, altering the correlation of ATP hydrolysis and Mre11 nuclease activity [34].

Long coiled-coil structures are shown to extend from the Mre11-Rad50 core (Fig. 2a). A recent crystallographic study reveals two rod-like human Rad50 coiled-coil dimers in which Zn^2+^ hook is located at apex (Fig. 2a) [35]. Two different assemblies, intermolecular and intramolecular complexes, were observed as a result of Zn^2+^ hook dimerization (Fig. 2e) [35–37]. Crystallographic studies on *Pyrococcus furiosus* Rad50 show that an octamer formed by two MR complexes extends in opposite directions from the Zn^2+^ hook dimer at the centre of coiled-coils (Fig. 2e, right). This result is supported by a later atomic force microscopy observation of a DNA-induced parallel arrangement of yeast MR complex, which together suggest the coiled-coils could act as a tether to bridge sister chromatids together [36, 38]. The intramolecular complex involving the dimerization of the Zn^2+^ hook at the apex of the coiled coil has also been proposed (Fig. 2a, e left). This model is consistent with the results that mutations of conserved Zn^2+^ hook cysteines impaired Zn^2+^ binding and dimerisation, but not sister chromatid cohesion [27, 37, 39, 40]. The predominant state in solution and their functional significances thus remain unknown.

Despite the significance advance, there is still a lack of structural information of the C-terminal domain of eukaryotic Mre11 which contains a glycine-arginine rich (GAR) domain, a region that a number of disease-associate variants are reported to be located [41, 42]. Furthermore, the structural information of the intact MRN, and how it recruits and regulates other DSB associated proteins, remains unknown.

## Structures of the large kinases that signal DDR

**Fig. 3:**
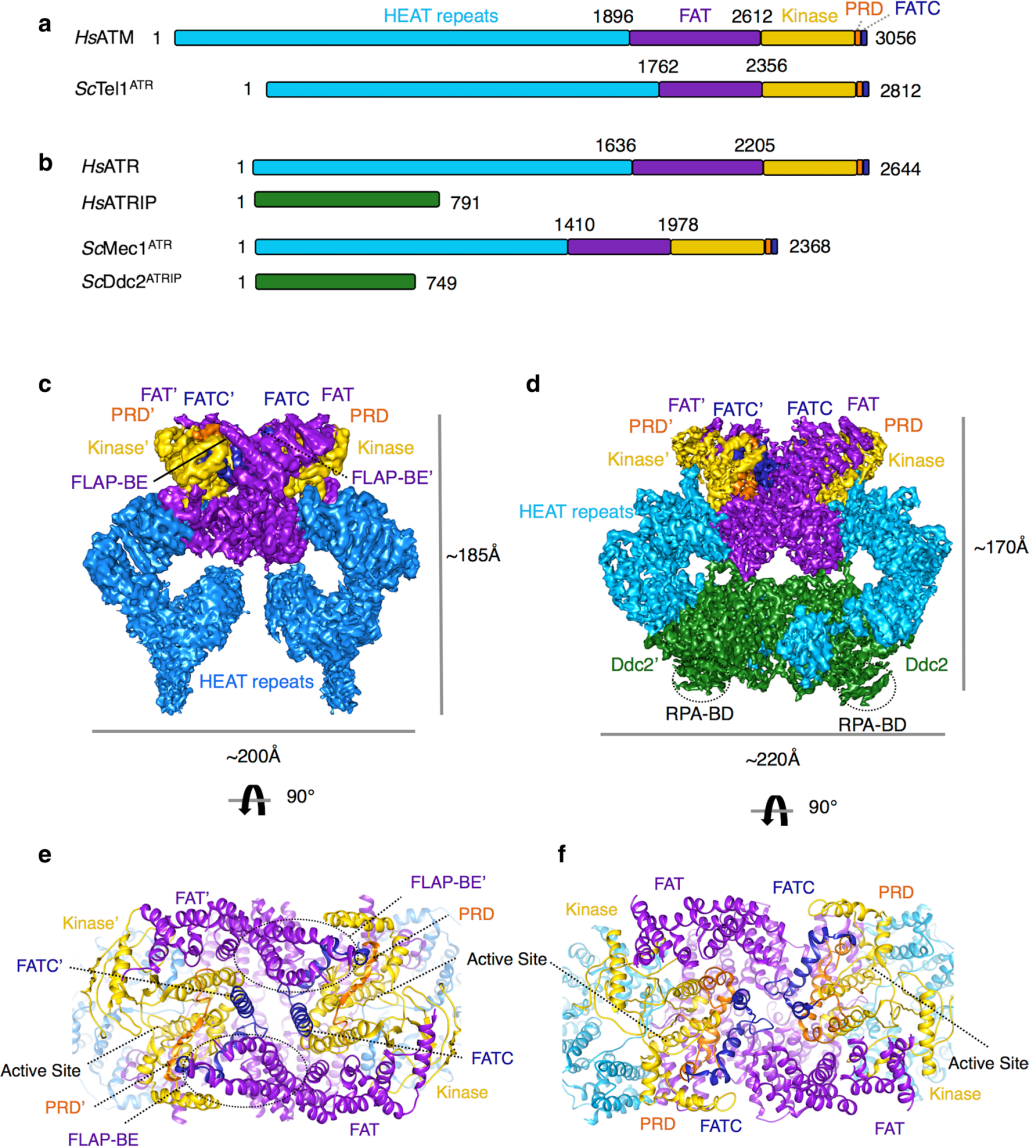
Structure of ATM and ATR-ATRIP. **a** Domain structure of human ATM (*Hs*ATM) and the *Saccharomyces cerevisiae* orthologue Tel1^ATM^ (ScTel1^ATM^) and **b** human ATR (*Hs*ATR) with its binding partner ATR-interacting protein (*Hs*ATRIP) with *Saccharomyces cerevisiae* Mec1^ATR^ (ScMec1^ATR^) and the ATRIP orthologue, Ddc2 (*Sc*Ddc2). The N-terminal HEAT-repeats are shown in cyan, the FAT (FRAP, ATR, TRAPP) in purple, the kinase domain in yellow, the PRD (PIKK regulatory domain) in orange and the FATC (FAT C-terminal domain) in dark blue. ATRIP/Ddc2, the specific ATR-interacting protein is also shown coloured green. Residue numbers are also labelled above. **c** The cryo-EM structure of human ATM and **d** the cryoEM structure of yeast Mec1^ATR^-Ddc2^ATRIP^, which is the most complete among the yeast and human structures, both coloured as in the schematic above, showing the dimeric architecture of these kinases along with approximate dimensions. **e**, **f** Features important for activity (see text), including the active site loop, are shown in a close up view of the atomic model, 90° rotated with respect to **c**, **d**

ATM, ATR and DNA-PKcs are key regulators of the DDR and perhaps some of the first transducers of damage signals. ATM, ATR and DNA-PKcs share structural similarities and domain organisations with other PIKK family members, such as mTOR (mammalian/mechanistic target of rapamycin) [43]. All PIKK family members share a conserved C-terminal PI3K-like kinase domain sandwiched between a unique region, N-terminal to the kinase, known as FAT (FRAP [FKBP12-rapamycin-associated protein], ATR, TRAPP [transformation/transcription domain-associated protein]) and, on the C-terminal side of the kinase domain, a PIKK regulatory domain (PRD) and FAT C-terminal domain (FATC). This catalytic portion only constitutes less than half of the polypeptide chain with the other comprising long stretches of HEAT (Huntington, elongation factor 3A, protein phosphatase 2A, TOR1) repeats. All PIKKs have between ~ 1400 and 3000 amino acid residues at the N-terminus that are arranged into these HEAT-repeat helical bundles described as a solenoid, that are likely important for recruitment of other proteins [44, 45]. ATR requires an integral partner, ATRIP, another HEAT-repeat containing protein (Fig. 3a, b). Due to the large size and limited sample quantity, these DDR PIKKs have been recalcitrant to traditional structural biology techniques such as X-ray crystallography, with the exception of DNA-PKcs and the C-terminal portion of mTOR [43, 46, 47]. More recently, with the advance of single particle cryoEM, there is a rapid increase in the number of low resolution and now higher resolution structures of ATM [48–52], ATR-ATRIP [53, 54] DNA-PK [44, 55] and mTOR [45, 56], providing significant insights into the precise domain organisation of these kinases. However, there is a clear lack of information on how these kinases are recruited and activated.

### ATM and ATR Structures

Several higher resolution (~ 4 to 9 Å) cryoEM structures have now been determined for the human ATM [50] and ATR-ATRIP [53], alongside structures of the yeast orthologues Tel1^A^™ [49] and Mec1^ATR^-Ddc2^ATRIP^ [54]. Overall, ATM and ATR-ATRIP adopt a similar “butterfly” architecture, and dimeric state with the kinase domain and tetratricopeptide repeat domains (TPR-D) of the FAT region forming a major dimerization interface [50, 53]. Additional dimer interfaces are also present in ATM and ATR contributed by the PIKK regulatory domain (PRD) and specifically for ATR/Mec1 an extensive interface contributed by ATRIP/Ddc2 dimerization along with the N-terminal region of the HEAT-repeats [53, 54] (Fig. 3c, d). DNA-PKcs differ in its quaternary structure as it exists as a monomer [47]. The kinase domain structure is highly conserved between the PIKKs, comprising a classical bi-lobed structure that binds ATP and magnesium ions in its cleft. The conserved activation loop, P-loop and catalytic loop are essential features for catalysis and are ordered in some of the high-resolution PIKK structures, which provides some insight into the regulation of the PIKK activity. Dimerisation of PIKKs has been described as a major mechanism for regulating the proper activity of such enzymes. The activation of these dimeric PIKKs has been proposed to rely on a dimer-to-monomer transition thus relieving the auto-inhibited state, resulting in a more accessible active site. Biochemical evidence for such a phenomenon is most compelling for ATM, with auto-phosphorylation at Ser1981 the switch for its activation [57]. ATR is also suggested to have an equivalent activation mechanism, with Thr1989 auto-phosphorylation the switch [58]. However, in both the ATM and ATR-ATRIP structures, the catalytic pockets of the kinase domain face away from each other. This outward facing arrangement, similar to mTOR, presents the active sites for substrate phosphorylation and does not explain how PIKK auto-inhibition and subsequent auto-phosphorylation occur, which is required for the activation of ATM and ATR [49, 50, 53, 54]. However, all the structures so far are in the absence of recruitment and activator factors, which might be required for a structural transition.

### Recruitment and activation of ATM

ATM is recruited to DSBs via its binding to the C-terminus of Nbs1 [2] (Fig. 1). The MRN complex can stimulate the activity of ATM directly in vitro [59], suggesting that the MRN complex is responsible for recruitment and activation of ATM. However, the exact details of how ATM is activated by MRN remain to be uncovered. The ATM cryoEM structures shed some light on its activation with two different structures captured. Two distinct populations of ATM dimer exist in solution and possibly in a dynamic equilibrium; a closely packed ‘inactive’ dimer, and a more open accessible active conformation [50]. In the ATM structure of the closed ‘inactive’ conformation, the FATC, PRD and activation loop form a closely packed structural feature denoted as the FLAP (FATC, LBE, activation loop and PRD) that associates with a helical hairpin from a TPR domain of the FAT region from the other monomer (denoted as the FLAP-binding element [FLAP-BE]) [50]. This intimate arrangement between the monomers reduces accessibility to the active site of either kinase domain due to a short region of the PRD being pushed into the active site pocket and the FLAP-BE occluding substrate binding [50]. In the ‘open’ dimer structure, determined from the same cryoEM dataset, the ATM dimer is asymmetric with one kinase domain rotated 24° with respect to the other, as compared to the “closed” dimer. This conformational difference reduces the extensive dimer interface and alleviates the restriction to the kinase active site by removing the interaction between the FLAP-BE of one monomer with the FLAP of another resulting in the PRD becoming disordered [50]. This equilibrium between a closed and an open ATM dimer would allow regulation from inactive to active by shifting the conformational equilibrium. This model is in agreement with earlier data showing that ATM is activated upon DNA damage by the acetylation of Lys3016 that sit close to one another and to the FLAP-BE in the closed dimer. Lysine acetylation, catalysed by the Tip60 complex [60], could weaken the dimer interface thus favouring an open active population. However, many questions remain, such as how ATM is activated by auto-phosphorylation, how is the equilibrium shifted by MRN and if a dimer to monomer transition occurs or is required for activation.

### Recruitment and activation of ATR

ATR has little basal activity and its activation is a multi-step process. In contrast to ATM and DNA-PKcs, which are activated by the recruitment of MRN and Ku70/80 complex to a DSB site, a range of genotoxic stresses elicit ATR signalling due to the fact that ATR, along with its obligatory binding partner ATRIP, is recruited to tracts of ssDNA that are coated with Replication Protein A (RPA) [61] (Fig. 1, see next section). These RPA-coated ssDNA recruitment platforms are typically generated as a result of stalled replication fork via the uncoupling of helicase and polymerase function, or the nucleolytic processing of DNA damage intermediates. It has also been observed that longer tracts of RPA-ssDNA serves as a more efficient platform for the recruitment of ATR-ATRIP [61]. The recruitment ATR-ATRIP (yeast Mec1-Ddc2) to RPA is mediated through interactions of an acidic patch (comprising Asp/Glu) of residues in the N-terminal domain of ATRIP/Ddc2 with a basic cleft of the N-terminal OB fold of the large subunit within RPA, RPA70 (and its yeast orthologue Rfa1) [61, 62]. The crystal structure of the N-terminal domain of Ddc2 (residues 1-109), which consists of the RPA binding domain (RBD) and coiled-coil domain (CCD), in complex with the Rfa1 N-terminal domain shows a mixture of hydrophobic and electrostatic interactions that drive the recruitment process [62]. The structure also shows that Ddc2 dimerises via its elongated CCD domain while each RBD domain interacts with a single Rfa1 N domain, consistent with earlier biochemical data of Ddc2 and ATRIP [3, 62, 63]. This crystal structure of the Ddc2 N-terminus is in stark contrast with the structural model proposed based on the 3.9 Å Mec1-Ddc2 cryoEM reconstruction, where a helical bundle domain, not directly involved in the dimer interface, has been built [54]. The crystal structure of Ddc2 is a domain in isolation whereas the cryoEM structure has limited resolution, thus the exact structures of Ddc2 and Mec1-Ddc2 complex require further investigation to reconcile these differences.

Unlike ATM with MRN, binding of ATR-ATRIP to RPA is insufficient to activate its kinase activity. Optimal ATR activation is achieved by the presence and interaction of additional activator proteins. The replication and DDR protein TopBP1 and its yeast orthologue Dpb11 are well characterised activators, which interact with ATRIP (Ddc2) and the PRD of ATR (Mec1) to stimulate the activation via its ATR-activation domain [64, 65], and can be recruited via its interaction with the damage clamp protein complex 9-1-1 (RAD9-RAD1-HUS1) (Fig. 1). A second ATR activator, ETAA1, was more recently discovered and is directly recruited via its interaction with RPA bound to ssDNA [64–68]. In budding yeast, alongside Dpb11, both Dna2 and Ddc1 (RAD9 in human) of 9-1-1 are Mec1/ATR activators. RPA and the 9-1-1 clamp are sufficient to recruit and activate Mec1 signalling in G1 phase and are required throughout the whole cell cycle. Dpb11 is additionally required in G2 phase whereas both Dpb11 and Dna2 are required in S phase [69]. Mec1 activation in yeast is mediated via two aromatic residues found in a long unstructured region of these activators [70]. Despite a wealth of biochemical and cellular work, the exact mechanism of ATR/Mec1 activation is still poorly understood. The recent cryoEM structures of Mec1-Ddc2 at 3.9 Å and the C-terminal catalytic core of ATR at 3.9 Å may partly explain the requirement of activators [53, 54].

In a similar fashion to ATM, the PRD plays an important role in ATR/Mec1 activation [64]. PRD is proposed to regulate dimerization and active site accessibility in both ATM and ATR/Mec1. The PRD of ATR/Mec1 forms an important dimer-interface of the global structure and this has implications on the conformation of the activation loop, P-loop and catalytic loop of the kinase domain. In the Mec1-Mec1 dimer, the PRD and FATC seem to hold and enclose the activation and catalytic loops of the kinase domain preventing access to the active site to substrates [54]. A key hydrophobic interaction between Met2312 of the PRD and Phe2244 of the conserved DFG motif in the activation loop has been shown to hold the kinase in an inactive state, with activators proposed to disrupt the PRD and alleviate the auto-inhibition [54, 64]. Intriguingly, the PRD is visible in the yeast Mec1 complex structure [54], but not in the human ATR complex, despite being at a similar resolution [53]. The structural insights thus far provide a framework to explain the low basal activity of ATR/Mec1 and the necessary requirement of activators to stimulate its kinase activity and why the PRD represents an important regulatory feature although the precise activation mechanisms requires the studies of activator bound complexes.

### RPA-coated ssDNA

**Fig. 4:**
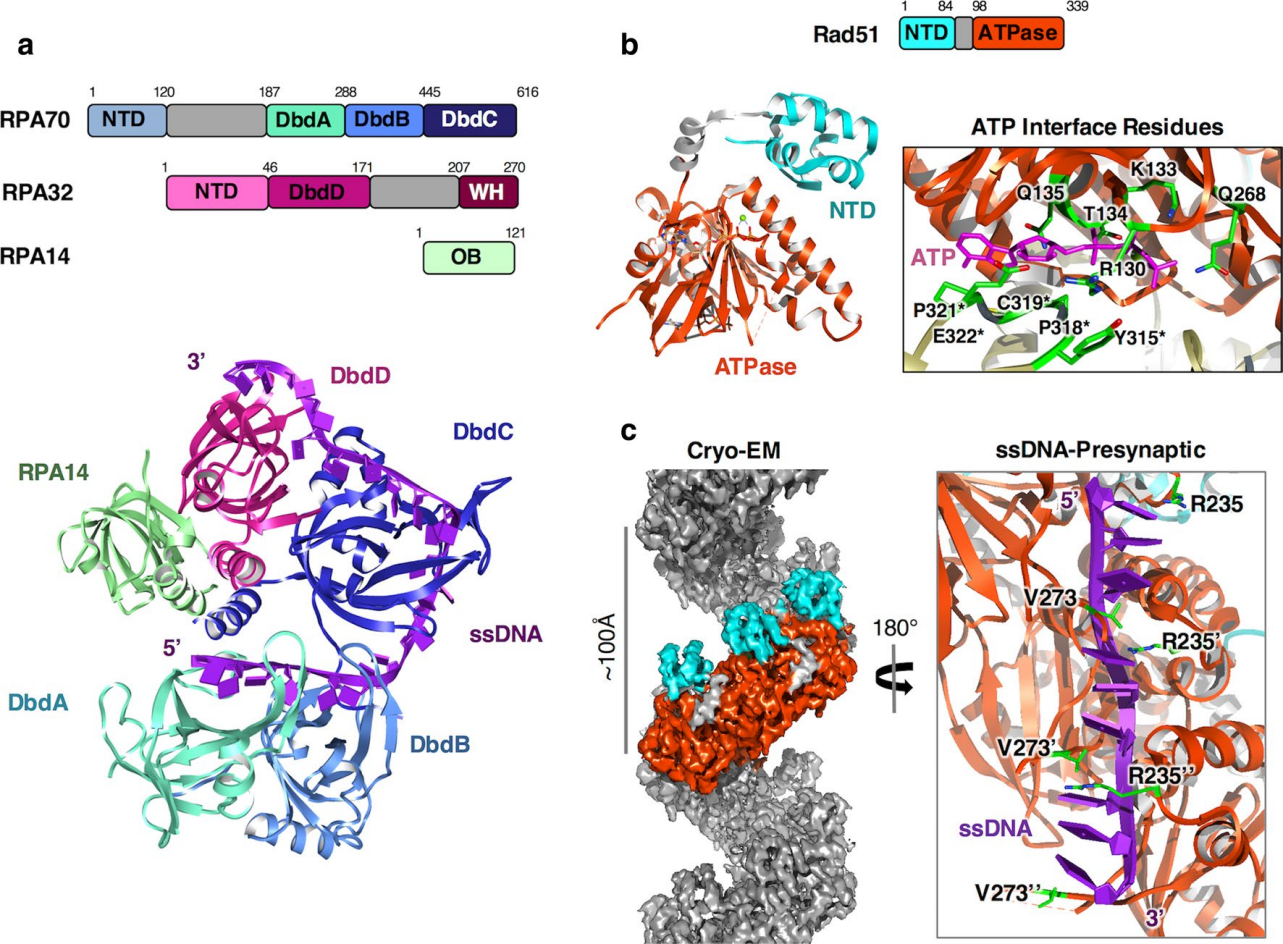
Known structures of RPA and Rad51. **a** Structure and domain organisation of RPA. RPA consist of three subunits: RPA70, RPA32, and RPA14. The structure shown is RPA from of *U. maydis* (PDB ID: 4GNX) showing secondary structure elements of the major OB folds involved in ssDNA binding. **b** Structure of human Rad51 in the presence of ATP showing secondary structure elements and the right-handed helical formation (PDB ID: 5NWL). Rad51 consists of a N-terminal DNA binding domain (residues 1–84), a small linker domain (residues 85–97), and a C-terminal ATPase domain (residues 98–339). Important residues involved in ATP binding are highlighted and are located at the dimer interface of two Rad51 monomers within the filamentous crystal structure. **c** CryoEM structure of the presynaptic Rad51 filament in the presence of AMP-PNP bound to ssDNA (EMDB ID: 9566, PDB ID: 5H1B). Due to the intercalation of Arg235 and Val273, the Rad51 filament engages DNA as triplet clusters extending the DNA length by nearly 1.5 times in comparison to B-DNA. This mode of binding may help in the formation of the synaptic filament during the search for homologous regions

The signalling at a DSB by ATM-MRN and further signalling by ATR-ATRIP generate long stretches of single-stranded DNA produced by DNA resection processes (dsDNA unwinding and nucleolytic digestion) mediated by Exo1, DNA2, and BLM-Sgs1 [71]. RPA plays significant roles both co-ordinating this process and simultaneously preserving the integrity of the resultant ssDNA. RPA is a ubiquitous heterotrimeric ssDNA binding protein that is essential to nearly all DNA processing events. Comprised of three protein subunits, RPA70, RPA32 and RPA14 (Rfa1, Rfa2 and Rfa3 in yeast), RPA contains multiple oligonucleotide/oligosaccharide (OB)-folds that interact with both ssDNA and proteins [72] (Fig. 4a). The large subunit, RPA70, contains 4 OB-folds; an N-Terminal Domain, DNA-binding domain-A [DbdA], B [DbdB] and C [DbdC] connected by flexible linkers. RPA32 possesses a single DNA-binding OB-fold [DbdD] with a long flexible N-terminal hyperphosphorylation region and a C-terminal Winged-Helix (WH) domain [73]. The smallest subunit RPA14 has a single OB-fold (DbdE). RPA associates with ssDNA with very high affinity via its core DNA binding domains DbdA, B, C from RPA70, and DbdD, from RPA32. The N-terminal domain of RPA70, as discussed earlier, and the winged-helix (WH) domain of RPA32 mediate the majority of the protein–protein interactions [74, 75]. RPA binds nucleic acids in two conformational states with different affinities for ssDNA; a lower affinity mode (dissociation constant Kd ~ 50 nM) occludes a binding site length of ~ 8 to 10 nucleotides [76–78] and involves DbdA and DbdB of RPA70 [76, 78–80], whereas a higher-affinity mode (Kd in the low nM—pM range) occludes 30-nucleotides and engages all four DNA-binding domains [76, 81]. This switch is coupled to a conformational change that has been captured by X-ray crystallography of *U. maydis* RPA [76]. The compact structure provides some clues to the coupling of RPA-ssDNA binding and allosteric coupling with protein recruitment, particularly in replication [76] (Fig. 4a). However, RPA is flexible [78], associates with ssDNA in many numbers and is heavily modified by ubiquitylation and phosphorylation during DDR [75]. The way in which multiple RPAs associate on ssDNA and coordinate its vast array of processes remains to be determined.

A crucial feature of RPA is that, whilst being able to bind nucleic acids with very high affinity, it must also be easily displaced to ‘hand-off’ ssDNA to other enzymes for further downstream processing. Single molecule work has shown that RPA can be displaced and exchanged with other ssDNA-binding proteins when there is sufficient concentration of unbound molecules [82]. This in vitro concentration-dependent exchange, referred to as facilitated exchange [83], is thought to rely on submicroscopic dissociated intermediates, where one of the DNA-binding domains is not bound to ssDNA, exposing a small patch of nucleic acid for other proteins to bind [82, 83]. However, this poses significant issues in vivo when considering inappropriate exchange, and there exists a number of proteins that facilitate the exchange of RPA for other genomic factors, such as Rad51.

## The Rad51 recombinase and its interactions with RPA

### Structure of Rad51 and nucleoprotein filament formation

The catalyst of DNA strand exchange and ultimately DNA repair is Rad51 recombinase. First discovered in yeast, it was recognised to be an orthologue of bacterial recombinase A (RecA) [84]. Recruited in an ATP-dependent manner to RPA coated single-strand DNA, mediated by BRCA2 in humans [85–87], this 43 kDa protein has been studied intensely since its discovery. Multiple crystal structures, EM reconstructions along with recent high resolution cryoEM structures have given much insight into its function. Rad51 has a two-lobed architecture of a mostly α-helical N-terminal domain of ~ 84 residues joined by a small helical linker to a larger C-terminal ATPase domain of ~ 240 residues [88–90] (Fig. 4b). Studies have described a DNA binding function to the N-terminal domain [91], possibly regulating the recognition of Rad51 for either ssDNA or dsDNA [92, 93]. The C-terminal domain contains a Walker A/B motif that is responsible for ATP binding and hydrolysis [94] (Fig. 4b). In the presence of ATP or ADP, Rad51 can readily form nucleoprotein filaments with DNA but the disassembly is dependent on the hydrolysis of ATP [95, 96]. Structures of the filamentous forms of the protein have shown that the ATP binding site is present at the interface between two Rad51 monomers [89, 90] (Fig. 4b). Residues Lys133 and Thr134 of human Rad51 from the Walker A motif bind to ATP where loop 315-323 from the adjacent monomer contributes hydrophobic interactions with the adenosine moiety. This stabilises interactions between Arg130 with Tyr315 and Glu322 from the adjacent monomer (Fig. 4b) and in turn communicates to residues involved in the binding of DNA such as Arg130 [97, 98].

Crystal and EM structures have also revealed the dynamic flexibility of Rad51 nucleoprotein filaments [89, 90, 99–102] where they vary in their pitch from 76 to 128 Å as compact and open forms respectively. This variation depends on the presence of DNA along with the state of the bound nucleotide. More specifically, filaments tend to be in more extended and dynamic state when ATP is bound versus when ADP is bound which might be an important feature in efficient homology searching [90, 100]. An example of this inherent flexibility is the recent crystal structure of human Rad51 filament in the presence of ATP where two different alternating dimer conformations were apparent which are due to small rotations around the ATP binding site [90]. Similarly the crystal structure of yeast Rad51 showed the presence of two different alternating dimer conformations around the ATP binding site which was due to a slight rotation between the N and C terminal domains [89].

Furthermore, high resolution cryoEM structures of the presynaptic (in the presence of ssDNA which represents the invading strand) and postsynaptic structures (in the presence of dsDNA, which represents the invading strand bound to a complementary strand) in the presence of AMP-PNP have shed light into the mechanism of DNA binding [101]. These structures show that Rad51 interacts with DNA through its phosphate backbone and very little structural changes occur when bound to ssDNA over dsDNA (Fig. 4c). In the presynaptic complex, each Rad51 monomer engages DNA in triplet clusters by the intercalation of Arg235 and Val273 (Fig. 4c). This mode is further stabilised by Arg235 interacting with the phosphate backbone of the complementary strand while having an electrostatic interaction with Asp274 when dsDNA is present. Hence this mode of DNA binding may be important in homology searching [101]. Though this study obtained a low resolution (~ 12 Å) structure of the synaptic complex and tentatively confirmed a second DNA binding site, much is still not known of how Rad51 binds dsDNA during strand invasion [101].

### Interactions with RPA and effects on Rad51 filament

During HR, Rad51 must somehow replace RPA on ssDNA (Fig. 1). This is predominately catalysed and stabilised by various mediator proteins that directly interact with both or either of these proteins (see next section) as RPA inhibits the loading of Rad51 on DNA [103]. Nevertheless the presence of RPA is important in stimulating strand exchange by Rad51 [104, 105]. As such it is interesting to note that Rad51 physically interacts with RPA, specifically between its N-terminal domain and the RPA70 subunit between residues 168-327, which contains DbdA (Fig. 4a) [106, 107]. This interaction is insufficient to fully displace RPA [107]. Recent single-molecule imaging experiments showed that the facilitated exchange of RPA allows the exchange with Rad51. However, free RPA in solution can inhibit Rad51 filament nucleation but has less effects on filaments elongation [83]. The dynamic exchange of RPA therefore facilitates Rad51 nucleation, but when sufficient amount of free RPA exists in solution, resulting from Rad51 nucleation/elongation, RPA then inhibits further nucleation. RPA therefore has a role in both facilitating and regulating Rad51 nucleoprotein filament formation.

### The Rad51 mediators

**Fig. 5:**
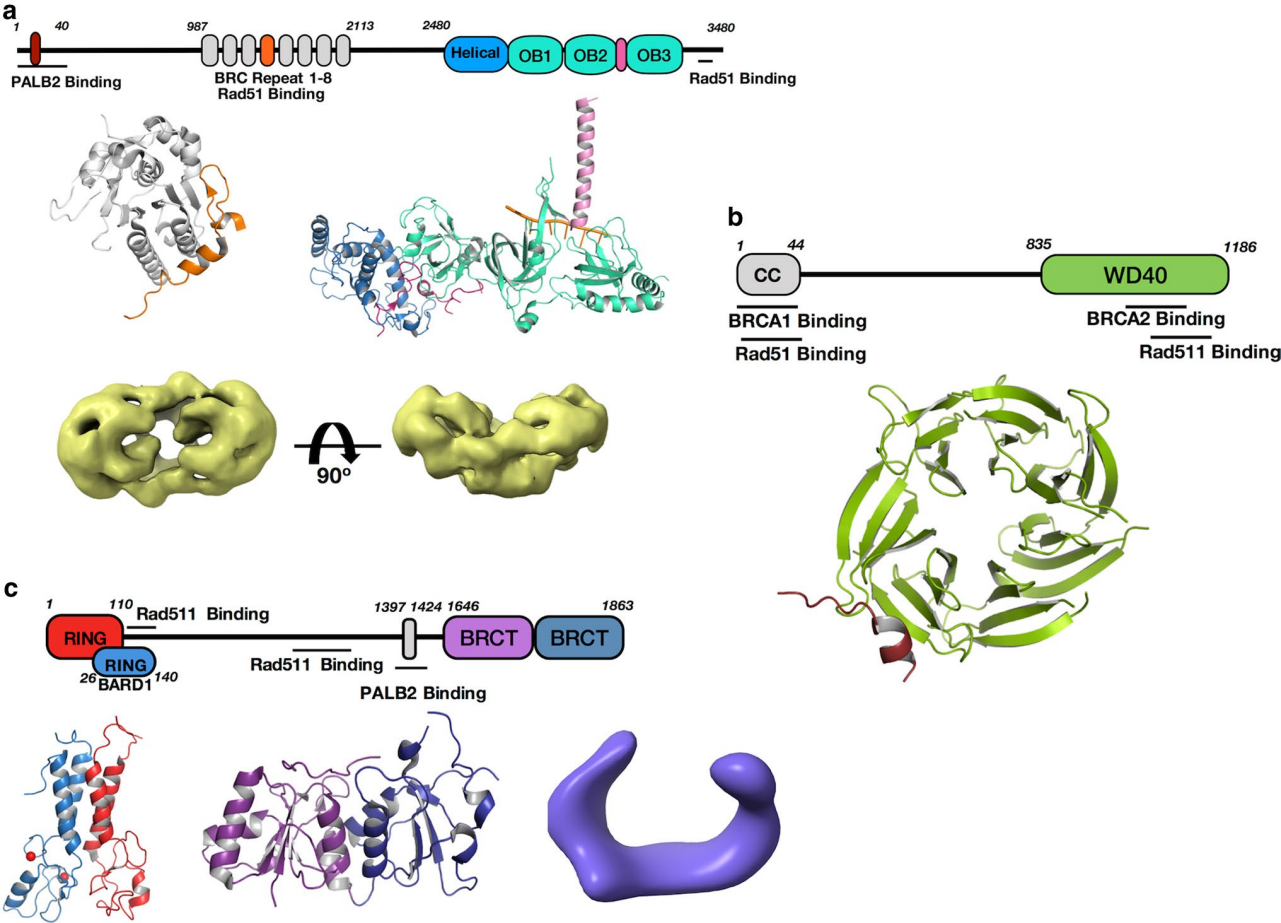
Known structures of the main Rad51 mediators BRCA2, BRCA1, and PALB2. **a** Domain structure of BRCA2. BRC4 motif is colored in orange in the BRC4-Rad51 complex (upper left, PDB ID: 1N0 W). Helical domain in BRCA2 C-terminal Domain (BRCA2DBD) complex with DSS1 and ssDNA (upper right, PDB ID: 1MJE) is colored in sky blue, three OB folds in cyan and tower domain in pink. Magentas coil near helical and OB1 represents DSS1. ssDNA is shown in orange. EM map of full length BRCA2 (bottom, EMDB ID: 2779) is colored in yellow with its orthogonal view. **b** Domain structure of PALB2 and crystal structure of its WD40 domain (PDB ID: 3EU7) bound to BRCA2 N-terminal motif is colored (ruby). **c** Domain structure of BRCA1. NMR structure of RING heterodimer of BRCA1 and BARD1, BRCA1 is colored in red and BARD1 in light blue, bound Zn^2+^ ions are shown in sphere representation (PDB ID: 1JM7). Crystal structure of tandem BRCT repeats (purple and blue, PDB ID: 1T29). EM map of BRCA1-BARD1 is shown in violet (EMDB ID: 8833)

The loading of RAD51 on 3′-overhanged ssDNA at DSB and formation of nucleoprotein filaments are highly regulated events, not just by RPA itself as discussed in the previous section, but by a number of dedicated mediators. The main mediator protein to load Rad51 on ssDNA is the BRCA2, a 3,418 residue protein. In cells, BRCA2 is recruited to DSB sites by Partner and Localizer of BRCA2 (PALB2) and BRCA1 and binds to ssDNA through its C-terminal DNA binding domain that contains several OB-domains (Fig. 5a). Only limited structural information of BRCA2 is available due to its large size, low natural abundance in cells and containing a large amount of intrinsically unstructured regions. Three crystal structures of various isolated domains and regions of BRCA2 have been reported: a BRC4 motif in complex with Rad51, a short N-terminal peptide in complex with the WD40 domain of PALB2, and a C-terminal helical domain followed by three OB-folds in complex with ssDNA and/or a small acidic protein DSS1 [88, 108, 109].

The C-terminal domain is the most conserved region of BRCA2. The crystal structures of ~ 800 amino acid C-terminal domain of mouse BRCA2 (BRCA2DBD) in complex with DSS1 and ssDNA determined more than 15 years ago [109], reveal an α-helical domain followed by three OB domains, forming a linear and elongated structure. The OB domains, both structurally and functionally, are similar to the OB domains in RPA as the BRCA2 OB domains can be replaced by RPA70 [110]. Both OB2 and OB3 have a canonical DNA binding groove accommodating the bound ssDNA. A long insertion in OB2 forms an α-helical coil which extends above and makes up the tower domain. The 70-amino acid DSS1 binds to BRCA2DBD in two sections with a disordered loop in the middle: the N-terminal portion of DSS1 tunnels through the helical domain and crosses the interface of OB1-helical domain whereas the C-terminal portion wraps around OB1 and ends up at OB1-OB2 interface. DSS1 is proposed as a ssDNA mimic that regulates both BRCA2-ssDNA and RPA-ssDNA associations. The latter is supported by biochemical studies demonstrating that DSS1 weakens the affinity of RPA toward ssDNA [111] and that BRCA2DBD-DSS1 complex simulates the displacement of RPA by Rad51 [111].

More recently, negative strain EM reconstructions of full length human BRCA2 and in complex with Rad51 have been reported [112]. In the reconstructions, BRCA2 dimerises to form an elliptical kidney-bean shape with C-terminal domains arching at both vertexes of the ellipse (Fig. 5a). Two sets of BRC repeats with bound Rad51 line up in the middle part. The dimeric BRCA2 is shown to bind to ~ 70nt ssDNA. It promotes RAD51 nucleation, but not filament growth. The structure provides insights into the overall architecture of intact BRCA2 as well as mechanism of Rad51 filament growth although the limited resolution precludes a precise assignment of structural domains [112].

In vivo studies suggest PALB2 recruits BRCA2 to nuclear foci and forms a complex with BRCA1 and BRCA2 [113–115]. However, apart from a C-terminal WD40 motif, a predicted chromatin binding motif and an N-terminal coiled-coil, which interacts with BRCA1, there is little structural information [113]. The structure of the WD40 domain in complex with the N terminal 18 amino-acid-peptide of BRCA2 shows that the BRCA2 peptide is accommodated at the outside of the β-propeller structure of the WD40 domain through polar interactions (Fig. 5b) [108]. The PALB2 binding motif on BRCA2 shows sequence conservation across species, suggesting the importance of the interactions in HR.

BRCA1 is a E3 ubiquitin ligase also involved in a diverse range of biological processes including HR [116–118], cell cycle checkpoint [119, 120] and transcriptional regulation [121, 122]. BRCA1 contains two major functional domains, the RING domain and tandem BRCT repeats at the N- and C-termini respectively (Fig. 5c) [123–125]. The RING domain at the N-terminus contains seven conserved Cys-His-Cys motifs with two bound Zn^2+^. Biochemical and NMR studies suggest it forms a tight heterodimer with BARD1, required for maintaining BRCA1 stability, E3 ubiquitin ligase activity and interaction with DNA [126–130], although its biological role and substrate in vivo are unclear [131, 132]. The C-terminal BRCT repeats incorporate two BRCT domains containing ~ 90 to 100 amino acids arranged in a linear fashion. The BRCT domains in BRCA1 play regulatory roles through the recognition of phosphorylated peptides with a pSXF motif on effectors such as 53BP1, BACH1, Abraxas, p53 and CtIP [123, 133–138]. The pSXF motif is recognised by a hydrophobic patch with a conserved lysine nearby. Similar features of BRCT domains have been observed in wide array of other DNA repair proteins, which bind to their respective physiological partner [139–141]. Although no high resolution structures of full-length BRCA1 are available, a low resolution negative stain EM structure of the BRCA1-BARD1 complex has been reported, in which it adopts a clamp-like architecture (Fig. 5c) [142].The RING domain and BRCT domain are mapped into the two opposite ends of the map. The presence of additional density near the RING domain is interpreted as ubiquitin in BRCA1 mutants.

### Interactions of mediators with Rad51

The main mediator for Rad51 loading on ssDNA is BRCA2 [85]. It interacts with Rad51 via its eight BRC repeats along with a small 36 amino-acid sequence at its C-terminus (Fig. 5a). The first four BRC repeats bind to Rad51 monomers with higher affinity and the binding inhibits Rad51 ATPase activity, thus indirectly enhancing Rad51 binding to ssDNA [143, 144]. The 5th–8th BRC repeats of BRCA2 preferentially bind to and stabilise Rad51 filament [86, 111, 143, 144]. Investigations of the various isolated BRC repeats along with a construct of all eight BRC repeats and the full-length protein have shown that they directly promote the binding of Rad51 onto ssDNA over dsDNA by both preventing ATP hydrolysis of Rad51, thus enhancing Rad51 binding to DNA and through the ssDNA binding of BRCA2 [143–146]. Furthermore, studies show that BRCA2 promotes Rad51 nucleation, thus a mediator role for BRCA2 has been proposed [112]. Besides the BRC repeats, another Rad51 binding site was reported at the C-terminal domain between 3265 and 3330 amino acid of BRCA2 (Fig. 5a) [147]. The interaction is regulated by CDK dependent phosphorylation on S3291. Loss of phosphorylation results in HR defect phenotype, speculating a regulatory mechanism of cell cycle check point on homologous recombination.

Structurally the BRC motif consists of ~ 30 amino acids that bind to the C-terminal ATPase domain of RAD51 (Fig. 5a). A β -hairpin structure is formed at N-terminus, leading to an amphipathic α-helix segment followed by a 3_10_ helix at the C-terminus. The hairpin extends the β -sheet on RAD51, mimicking the monomer–monomer contacts in a RAD51 filament [99]. BRC4 thus inhibits RAD51 oligomerisation. The ATPase active site is also inhibited allosterically through a BRC4 induced conformational closure to preclude ATP binding. The conformational change would also expose the BRC binding sites on adjacent promoters in a Rad51 filament/oligomer (Fig. 5a) [88, 148, 149].

It is also of note that the BRCA2 interacting proteins of PALB2 and the BRCA1-BARD1 complex have also been reported to bind RAD51, however no evidence of BRC repeats are present in their sequences [150–152]. PALB2 has been shown to bind Rad51 at both its N and C termini (residues 1–200 and residues 853–1186). On the other hand, Rad51 has been shown to bind to the centre of BRCA1 (residues 758–1064) and near the N-terminus of BARD1. Both PALB2 and the BRCA1-BARD1 complex have been reported to stabilise Rad51 nucleoprotein filaments during recombination and promote strand exchange [150–152]. As PALB2 is the bridging protein to form a complex between BRCA1 (possibly BARD1) and BRCA2 [113, 153], these findings imply that it is possible that all components of the resulting complex are involved in the loading and stabilisation of Rad51 on ssDNA (Fig. 1). However, further information is required on the exact roles of these proteins and more importantly, on the mechanisms of their actions.

A number of other complexes have also been reported to promote and stabilise the Rad51 filament during homology searching and strand invasion (Fig. 1) [154]. Two such complexes are Rad51 paralogues complexes, BCDX2 (Rad51B-Rad51C-Rad51D-XRCC2) and CX3 (Rad51C-XRCC3), though they have other roles earlier in the process of homologous recombination [155–157]. These paralogues have 20–30% sequence homology with RAD51, share a similar domain structure, [155], interact with Rad51 [158], bind to DNA [159], and are essential for homologous recombination [160]. Another complex involved in maintaining the Rad51 filament is the Shu complex in yeast consisting of Shu1, Shu2, Psy3, and Csm2 [161, 162]. A recent crystal structure of the complex revealed a V-shaped architecture and showed that Shu1, Psy3, and Csm2 are Rad51 paralogues, whereas Shu2 formed a novel fold with a zinc-finger domain [163]. In humans SWS1 and SWSAP1 have been identified as homologues of the Shu complex and are likely to have a similar role [163–165].

## Future perspectives

Although structural and biochemical studies have revealed a plethora of information on the various roles of major players within the homologous recombination pathway, it is still unclear how their activities are regulated and coordinated. Due to the large size and difficultly in obtaining these proteins recombinantly and in assembling stable and homogenous functional complexes [85–87], structural insights have been elusive, especially for larger complexes with multiple components. However, with the recent advance in cryoEM [166] and the ability to produce recombinant complexes [167], we envisage that structural insights of these important complexes should be attainable in the near future.

## Abbreviations

ADP: Adenosine diphosphate
AMP-PNP: Adenylyl-imidodiphosphate
ATM: Ataxia telangiectasia mutated
ATP: Adenosine triphosphate
ATR: Ataxia telangiectasia and Rad3-related protein
BARD1: BRCA1-associated RING domain protein 1
BRCA1: Breast cancer type 1 susceptibility protein
BRCA2: Breast cancer type 2 susceptibility protein
BRCT: BRCA1 C-terminal domain
cryoEM: Cryo electron microscopy
DDR: DNA damage response
DSB: Double-strand break
EJ: End joining
MRE11: Meiotic recombination 11 homolog 1
MRN: MRE11 RAD50 NBS1
NBS1: Nijmegen breakage syndrome protein 1
OB: Oligonucleotide/oligosaccharide-binding
PALB2: Partner and localiser of BRCA2
RPA: Replication protein A
